# The House: Its Unique Problems in Hygiene

**Published:** 1915-09

**Authors:** 


### THE HOUSE: ITS UNIQUE PROBLEMS IN HYGIENE.

A house erected in accord with modern science and the builder's art must satisfy a few apparently simple needs. These have been cleverly summarized in the following words by Kinne, Helen, and Cooley, Anna M., in Shelter and Clothing, New York, 1914, p. 15: Protection from the elements, from cold and heat, from rain and snow and damp, from intruders who might interfere with the family safety or possessions; water at hand; some way of getting rid of waste; space for the family, for all their occupations and belongings; room for a guest. These were sought by even the cave dwellers. And we have not passed beyond these simple needs. Our enemies are of a different kind, but the daily paper shows that we must pay for safety locks; and while wild animals no longer prowl about, we find it almost impossible to keep out rats and mice and harmful insects. The "house" fly is now called a "typhoid" fly, and not permitted even as a casual visitor. To all these needs we have added what the cave man did not seek for, since his life was largely out of doors. We must have air and sun within doors. Doctors are now talking about house diseases. Tuberculosis is one of these, and the fight against it must be made, in part, just here. It is for sun and air that we have to pay large rents in town; and it is partly to secure these in our large dwellings that tenement-house commissions exist, to protect those who can not protect themselves. Then, too, there must be protection against fire, not only by the fire department but also in the house itself. Modern nerves, moreover, demand quiet. We may want our own phonograph, but we do not care to hear our neighbor's, and walls and floors must be built to keep out sounds. We call these simple needs. They would seem to be human rights; but even now, in this twentieth century, how many houses rank 100 per cent. in all these; in warmth and coolness at proper seasons; perfect dryness, ventilation and lighting; safety from fire and intruders; and room for each member of the family to be by himself, and to keep an open door to guests? Yet, we can not be as well or as happy or as useful as we should, until these are achieved.

Only a few years ago, such subjects were regarded by some as beneath the dignity of scientific investigation. Miss Helen W. Atwater, of the Office of Experiment Stations, remarks that the way in which our homes are run, or, in more technical terms, the science of home economics, is now in much the position that scientific agriculture was in twenty or thirty years ago. The leaders had shown that science can improve crops, and some of the more progressive farmers were giving the new ideas a practical test; but many of the rank and file were still doubtful whether such study was worth while. Few farmers of today, however, would care to go back to the days before experiment stations, fertilizer control and other modern improvements. The creation of a home, to be as efficient as possible, includes many different kinds of questions. It makes necessary much study along many lines, just as agriculture has included problems as different as those of insect pests and cheese making. In solving agricultural problems, every intelligent farmer has done his part as well as the special investigators in the laboratories. In the same way, every intelligent housekeeper who studies the household problems of cooking, cleaning and furnishing, and tries to solve them with the help of both practical experience and scientific information, hastens the day when household management can be as accurately planned as that of the factory and the farm.

There are many features of the situation which appeal to the physician. He is concerned with all the modern questions of industrial hygiene, the dangers of occupations, and the harm which may come to mankind as a direct result of environment and mode of work. In a sense, the house must be considered as a workshop. It is "sometimes a bakery, sometimes a clothing factory, sometimes a cleaning establishment." The house, therefore, should be equipped for efficiency in carrying on housework just as carefully as a modern shoe factory is equipped for making shoes. In such a factory, lighting, heating, ventilation and sanitation are as carefully considered as the machinery. These matters of hygiene are even more important in the home, which is not merely a workshop, but also a place in which to rest and recuperate.

Ridicule or accusations of extreme faddism no longer greet those who advocate a rational dietary. There is a growing tendency to study the intelligent expenditure of scanty portions of small incomes, so that the purchase of potatoes instead of pickles, and of wholesome food calories instead of advertised dainties and delicacies, is becoming less unusual. It will not be long before a frank discussion of the economy of other features of the home will be more freely tolerated. The rational use of labor-saving devices not only may bring comfort but also may avert difficulties that bring patients to the physician. As Miss Atwater remarks, a machine for washing clothes saves more bodily energy than a patent roasting pan, and a meat chopper is used more often than a device for stoning cherries.

There are plenty of problems bordering on the domain of industrial hygiene in relation to household equipment and management.—*Journal A. M. A.*, August 28, 1915.

